# On the origin of microbial ORFans: quantifying the strength of the evidence for viral lateral transfer

**DOI:** 10.1186/1471-2148-6-63

**Published:** 2006-08-16

**Authors:** Yanbin Yin, Daniel Fischer

**Affiliations:** 1 Computer Science and Engineering Dept. 201 Bell Hall, University at Buffalo, Buffalo, NY 14260-2000, US; 2 Bioinformatics/Dept. of Computer Science, Ben Gurion University, Beer-Sheva 84015, Israel

## Abstract

**Background:**

The origin of microbial ORFans, ORFs having no detectable homology to other ORFs in the databases, is one of the unexplained puzzles of the post-genomic era. Several hypothesis on the origin of ORFans have been suggested in the last few years, most of which based on selected, relatively small, subsets of ORFans. One of the hypotheses for the origin of ORFans is that they have been acquired thru lateral transfer from viruses. Here we carry out a comprehensive, genome-wide study on the origins of ORFans to quantify the strength of current evidence supporting this hypothesis.

**Results:**

We performed similarity searches by querying all current ORFans against the public virus protein database. Surprisingly, we found that only 2.8% of all microbial ORFans have detectable homologs in viruses, while the percentage of non-ORFans with detectable homologs in viruses is 7.9%, a significantly higher figure. This suggests that the current evidence for the origin of ORFans from lateral transfer from viruses is at best weak. However, an analysis of individual genomes revealed a number of organisms with much higher percentages, many of them belonging to the Firmicutes and Gamma-proteobacteria. We provide evidence suggesting that the current virus database may be biased towards those viruses attacking Firmicutes and Gamma-proteobacteria.

**Conclusion:**

We conclude that as more viral genomes are sequenced, more microbial ORFans will find homologs in viruses, but this trend may vary much for individual genomes. Thus, lateral transfer from viruses alone is unlikely to explain the origin of the majority of ORFans in the majority of prokaryotes and consequently, other, not necessarily exclusive, mechanisms are likely to better explain the origin of the increasing number of ORFans.

## Background

ORFans are defined as ORFs (Open Reading Frames) having no sequence homologs in other genomes [1]. ORFans with homologs in the same genome are called paralogous ORFans, and those without any homolog whatsoever are called singleton ORFans. In addition, orthologous ORFans are defined as those ORFs with homologs only within very closely related microbial genomes [2]. Nearly all the fully sequenced genomes have a significant number of ORFans, although the percentages in different species vary much. Previous studies in our group [3], and subsequently repeated by others [4] have shown that as more genomes are being sequenced, the number of ORFans continues to grow. Despite their abundance, very few ORFans have been experimentally characterized [5-7], and thus, most ORFans in the database are annotated as hypothetical proteins of unknown function.

Since we coined the term "ORFan" in 1999 [1], accumulating evidence has demonstrated that most ORFans correspond to real, functional proteins, and not to errors in the ORFs annotation [5,7-10]. A survey of ORFans whose three dimensional structure has been determined [11] suggested that ORFans are already being studied by a number of groups. In addition, base compositional analysis and Ka/Ks tests on ORFans conserved in closely related species (orthologous ORFans) have shown that at least some of them are real, functional, proteins, although many may have lower GC content and appear to evolve faster than widely conserved genes [9,12-14].

Because of the lack of homology to other proteins, the origin of ORFans entails an evolutionary puzzle. Recently, a number of hypothesis regarding their origins have been proposed. The observation of the different sequence characterization and phylogenetic distribution of poorly conserved ORFans suggested that they may correspond to laterally transferred genes (LTGs) [12]. This presumption was extended recently in a study of Gamma-proteobacteria which proposed that ORFans are likely to be LTGs from viruses [9]. The most direct evidence that can support the hypothesis that ORFans originate from viruses is to find homologs to these ORFans within the virus sequence database. Daubin and Ochman BLASTed selected ORFans from *Escherichia coli *MG1655 genome against sequenced bacteriophage genomes and found that only ~9% of the ORFans have homologs in phages [9]. This was an unexpectedly low percentage to give strong support to the hypothesis, but the authors explained that the low number may be due to the very limited sampling of virus sequences [9,15,16]. In the past year, this hypothesis and explanation have further been developed [17-21].

However, the supporting evidence for this hypothesis and its subsequent explanation of low sampling was based on observations from various types of ORFans (restricted in different depths of phylogeny) within Gamma-proteobacteria only [9]. Here, we carry out a genome-wide study aimed at quantifying the strength of this hypothesis, focusing only on singleton, paralogous and very narrowly defined orthologous ORFans. Our datasets include the 277 microbial genomes and the 1456 viral genomes available on November, 2005.

## Results

### ORFan collections and the three categories of ORFans

Our collection of ORFans from 277 genomes included the three types of ORFans: singleton ORFans (ORFs with no homology to any other protein), paralogous ORFans (ORFs with homology to proteins of the same genome only) and orthologous ORFans (ORFs with homologs in closely related organisms only). Identifying the first two types of ORFans is straightforward using BLASTP (see Methods). However, to automatically collect orthologous ORFans, an operational definition of "closely related organisms" is needed [22]. To this end, we developed a novel method to define two featured values for each microbial ORF. The first is the "H value", which is simply the number of genomes (including the residing genome) that contain at least one homolog of this ORF. The values of H are integers in the range from 1 to N, where N is the number of genomes considered (277 here). So an ORF with H = 1 corresponds to either a singleton ORFan (the only BLASTP hit is to itself) or to a paralagous ORFan (more than one BLASTP hit, but all within the residing genome). The second value we define is the "U value", which is a measure of the "uniqueness" of the ORF, and is a normalized sum of the ORFs homologs, weighed by the overall genomic distance between the residing genome and the genomes of the ORF's homologs (see Methods). The values of U range between 0.0 to a theoretical maximum 1.0. Thus, ORFs with H > 1 and very small U correspond to ORFans having homologs in closely related genomes only.

However, choosing a cutoff value of U to define orthologous ORFans is not straightforward. Based on observation, we (rather arbitrarily) set the U value cutoff at 0.1. For example, using this threshold, we consider the hypothetical protein SA2483 (gi: 15928277) from *Staphylococcus aureus *strain N315 as an orthologous ORFan, with U = 0.099 and H = 5. SA2483 has homologs in four other genomes, all within the *Staphylococcus *genus: *S. saprophyticus*, *S. haemolyticus*, and two *S. aureus *strains: Mu50 and COL. Another example of an orthologous ORFan is hypothetical protein b1407 (gi: 16129368) from *Escherichia coli *strain K12, with U = 0.092 and H = 7. It has highly similar homologs in *E. coli *O157H7 strain EDL933 and *E. coli *O157H7, and in two *Shigella *genus genomes: *S. flexneri 2a srtain *2457T and *S. flexneri 2a srtain *301. In addition, its C terminus is about 40% identical to a paralogous ORFan in *E. coli *strain K12 and to ORFans in other *Escherichia *and *Shigella *genus genomes.

**Figure 1:**
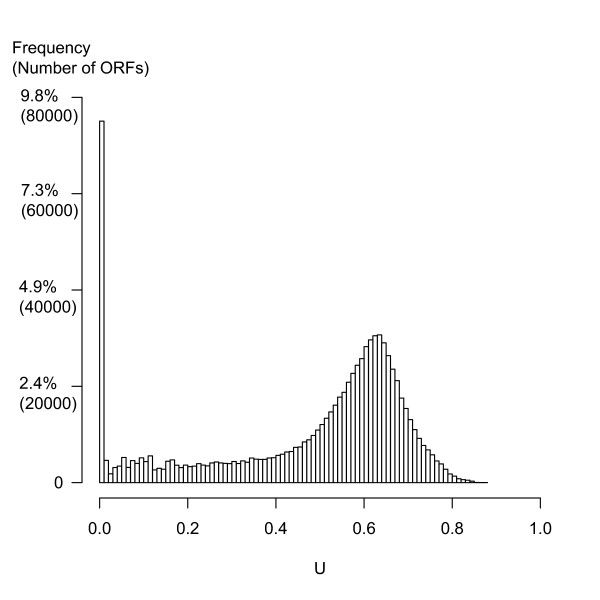
U-value histogram for all the 818,906 ORFs in 277 prokaryote genomes. The U-value is a measure of the "conservation" of each ORF (see Methods); U = 0 means the ORF is unique to one single organism, i.e. a singleton or paralogous ORFan. 9.1% of all ORFs have U = 0. The left tail 0.0 < U <= 0.1 (4.3% of all ORFs) corresponds to orthologous ORFans, ORFs with homologs only in closely related organisms. Notice the uneven distribution of U, with its long left tail and the very high peak at U = 0.

Figure 1 shows the histogram of U values for all 818,906 ORFs in the 277 genomes. The histogram shows a peak value at 0.64, and a long left tail, the end of which corresponds to our definition ORFans (orthologous ORFans at 0.0 < U <= 0.1 and singleton and paralogous ORFans at U = 0.0). In total, we collected 110,186 ORFans (13.4% of all ORFs in the 277 genomes) of which 64,324 (7.8%) are singleton ORFans, 10,419 (1.3%) are paralogous ORFans and 35,443 (4.3%) are orthologous ORFans (also see Additional file 1). These ORFan percentages are similar to those previously reported using 60 [3], 122 [4] and 127 genomes [12]. In summary, using the U and H measures we can automatically and objectively compile our collection of the three types of ORFans.

### Percentage of microbial ORFs having viral homologs

**Figure 2:**
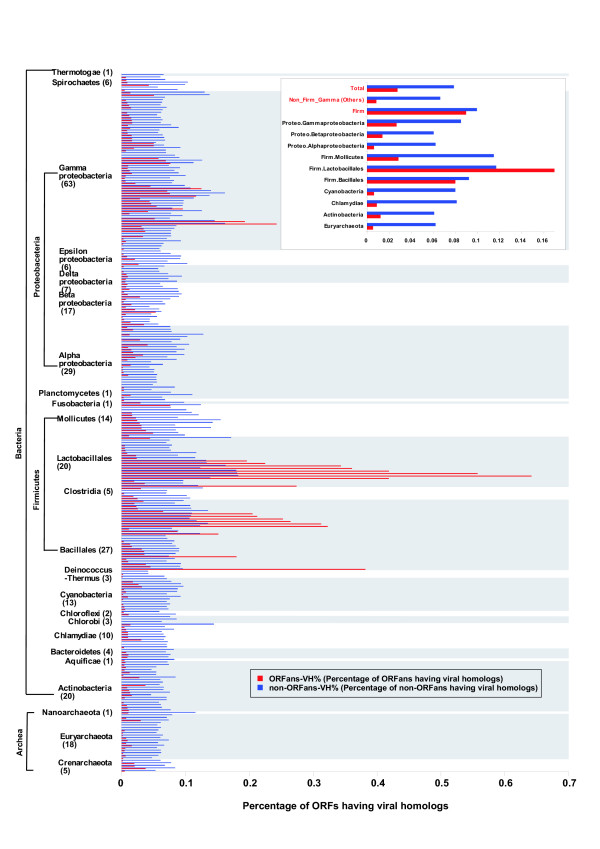
Percentage of microbial ORFs having homologs in viruses for 277 prokaryote genomes. The y-axis shows each of the 277 genomes, grouped according to NCBI's taxonomy classification. For each genome, two percentages are shown: red corresponds to ORFans-VH% (percentage of ORFans having homologs in viruses) and blue corresponds to non-ORFans-VH% (percentage of non-ORFans having homologs in viruses). The major 24 clade names are shown, with the number of organisms in each clade shown in parenthesis. The 24 phylogenetic clades are alternately marked by grey and no background colors. For the species names and taxonomies, please refer to Additional file 1. The inset shows the average percentage values of ORFans-VH% and non-ORFans-VH% in various groups. "Total" corresponds to the averages in all 277 genomes taken together, "Non_Firm_Gamma (Others)" corresponds to the averages in the 148, non-Firmicutes, non-Gamma-proteobacteria genomes and "Firm" corresponds to the 66 Firmicutes in the database. The remaining groups in the inset correspond to the major clades (with at least 10 genomes). The figure clearly shows that except for some Firmicutes, ORFans-VH% is much smaller than non-ORFans-VH%, suggesting that the current evidence from homology supporting the hypothesis that the origin of ORFans is viral is weak at best.

For each proteome in our 277 genome database, we performed a BLASTP search against the public virus proteins database and computed two percentages: ORFans-VH%, the percentage of ORFans having homologs in viruses and non-ORFans-VH%, the percentage of non-ORFans having homologs in viruses. Figure 2 shows for each genome the computed ORFans-VH% and non-ORFans-VH%. The genomes are taxonomically clustered, so that closely related genomes appear close to each other in the figure. The figure shows that ORFans-VH% varies much (range: 0~63.8%) in different microbial genomes, while non-ORFans-VH% varies much less (range: 4.1%~18.2%).

One way to quantify the strength of the hypothesis that the origin of ORFans is viral is to compare the value of ORFans-VH% with that of non-ORFans-VH%. A significantly higher ORFans-VH% would suggest that viral transfer is more common among ORFans than among non-ORFans (which corresponds to the overall detectable baseline of transfer). Surprisingly, we found that out of the 277 genomes studied, only 22 (7.9%) had ORFans-VH% > non-ORFans-VH%. Eighteen of these 22 genomes are members of Firmicutes, and 4 belong to Gamma-proteobacteria. The highest value of ORFans-VH% is 64% in *Streptococcus pyogenes *MGAS315 (with 154 of its 240 ORFans having homologs in viruses; see Fig. 2 and Additional file 1). The highest number for non-ORFans-VH% is 18% (also for *S. pyogenes *MGAS315). On the other hand, 44 (15.9%) genomes have ORFans-VH% = 0 and non-ORFans-VH% significantly lower than that of the rest of the genomes (Wilcoxon nonparametric p-value 9.1e-05). These low percentages are not a result of very low numbers; most of these genomes have more than 100 ORFans, comprising between 3% and 29% of all ORFs in the genome. Taking all the genomes together we found that only 2.8% of all ORFans have viral homologs while the percentage for all non-ORFans is 7.9% (p-value 2.2e-16). These findings suggest that the evidence based on current homology to viruses is very weak in general.

Significant differences are also observed when comparing the non-ORFans-VH% versus the ORFans-VH% values of various groups (see inset of Fig. 2). Firmicutes (66 genomes) and Gamma-proteobateria (63 genomes) have significant higher percentages than the other 148 genomes ("Others"). The values for non-ORFans-VH% and ORFans-VH% are: Firmicutes: 10.0% and 9.0%, Gamma-proteobacteria: 8.5% and 2.7%, Others (148 genomes): 6.6% and 0.8%; (see Fig. 2 inset). These differences are statistically significant (p-values for ORFans-VH%: firm vs. others: 7.8e-13, gamma vs. others: 6.6e-08, firm vs. gamma: 7.8e-03; and p-values for non-ORFans-VH%: firm vs. others: 2.2e-16, gamma vs. others: 5.8e-05, firm vs. gamma: 1.3e-05). These figures show that Firmicutes and Gamma-proteobacteria have the highest number of homologs in viruses (both ORFans and non-ORFans), and that for genomes in the "Others" group, the number of ORFans with virus homologs is negligible (ORFans-VH% = 0.8%).

To check whether the above numbers are a result of stochastic effects caused by short ORFs, we changed the cutoffs defining ORFans (ORF length longer than 300 bp or E value less than 1e-10; see Methods). All the above observations remained significant (Additional file 1). In addition, to check whether the percentages may be skewed for genomes with very few total ORFans, we recomputed the percentages after removing the genomes with less than 100 ORFans and the above observations also remained significant (data not shown).

## Discussion

The abundance of ORFans observed today in the genetic material has become one of the unexpected surprises in the post-genomic era. Their functions and origins remain unresolved puzzles. One of the hypotheses regarding the origin of ORFans, derived from a limited analysis in Gamma-proteobacteria only, suggests that microbial ORFans are of viral origin, transmitted by phages [9]. In this paper, we carried out a genome-wide study to attempt to quantify the strength of the currently available evidence that supports this hypothesis.

By searching strictly defined ORFans from 277 microbial genomes against the public viral protein database, we found viral homologs for only 2.8% of the ORFans. This suggests that transfer from viruses can today account for only a tiny fraction of the ORFans. Furthermore, negative support to the hypothesis is obtained when comparing the percentage of ORFans with viral homologs (2.8%) with that of non-ORFans (7.9%), because this suggests that transfer from viruses is significantly less frequent for ORFans than for non-ORFans. Out of the 277 genomes, we found only 22 genomes with a percentage of ORFans having viral homologs higher than that of non-ORFans. Eighteen of these genomes belong to the Firmicutes and the other 4 belong to Gamma-proteobacteria. These findings partially explain Daubin and Ochman's hypothesis which was mainly based on analysis of one of these clades but show that the strength of the current evidence for viral origin for ORFans in general is very weak at best.

Nevertheless, the weak evidence of homology to viral proteins does not necessarily imply that the hypothesis is wrong. As Daubin and Ochman argued, the low percentages observed today may be due to the current extremely low sampling of virus sequences [9]; while the current database contains ~10^3 ^viral genomes, it is estimated that the virus population size in the ocean alone is ~4 × 10^30 ^[23], and more importantly, that the phage diversity is ~10^8 ^[24]. Thus, due to this huge diversity, it is plausible that a significant fraction of the ORFs without detectable viral homologs today may have originated from not yet sequenced or extinct viruses.

In addition, the significantly higher percentage of microbial ORFs with viral homologs in Firmicutes and Gamma-proteobacteria may indicate that the current virus database is biased to contain more viruses attacking these two clades. To test this, we first computed the host distribution of the viral genomes by parsing the Genbank format files of the phage genomes in our database and manually checked the related literature to determine what specific host each phage infects and what taxonomical group each microbial host belongs to. Of the 1456 viruses in our database, 280 are phages. We found that 109 phages target Gamma-proteobacteria, 102 target Firmicutes and 69 target "Others". We also computed the number of viral genomes containing homologs in each of the three prokaryotic genome groups. Then, for each group, we divided this number by the number of genomes in the group. We found that Gamma-proteobacteria and Firmicutes have higher average ratios (4.84 and 4.68, respectively) than the "Others" group (2.53), and a statistical test considering the number of viral genomes containing homologs for the individual genomes in each group showed the differences are statistically significant (all pairwise p-values << 0.01). These two results suggest that the current viral databases are indeed biased towards viruses attacking these two clades. Furthermore, because the overall percentage of ORFs (ORFans and non-ORFans) with viral hits in Gamma-proteobacteria is lower than that of Firmicutes (see above), but both our host count and computed average ratios for Gamma-proteobacteria and Firmicutes are approximately the same, it seems that the current database contains a further bias towards Firmicutes in general.

Thus, we can only claim that the evidence today is weak in general, and future sampling of the viral genomes may provide stronger evidence. With more viruses sampled, at least for some prokaryote genomes, the percentage of ORFans with homologs in viruses can become very high. Thus, for some clades, further viral sampling will likely provide stronger evidence for the viral origins of ORFans. However, it is questionable whether a full sampling of viral genomes will provide homologs to 100% of the ORFans or only to a fraction of them.

In addition, even better sampling of viral genomes may not allow explaining the presence of ORFans in some of the microbial genomes. We have found 44 genomes with no viral homologs for any of their ORFans, and with a percentage of non-ORFans with virus homologs significantly lower than the rest of the genomes. This suggests that they may correspond to genomes immune to viral attack, as is the case for obligate species; indeed, 21 of the 44 genomes are classified as pathogens or symbionts according to the organism info page at [25]. Because their hosts are a natural barrier to prevent attack from phages, lateral transfer is rare or non-existent among obligate symbionts and pathogens [26,27] (and references therein). Consequently, it is to be expected that they show lower percentages of (ORFan and non-ORFan) viral homologs. Thus, unless the ORFans in these genomes were acquired from viruses before the organisms became host-dependent, their origin is not likely to be from viral lateral transfer, but rather from alternative, yet unknown, mechanisms.

Independently of the amount of viral genome sequences sampled, many ORFans may remain without viral homologs. This could still be compatible with the possibility of ORFans having viral origins, if it is assumed that ORFans have experienced rapid evolution [9,12,28] after being transferred from viruses. Their sequences may have diverged to the extent that no homology to viral proteins is detectable, and some of them may have even acquired novel functions or three-dimensional structures [11]. Although this is a plausible explanation of the origins of some of the ORFans, further genome-wide studies may be required to quantify the strength of the evidence for this hypothesis.

## Conclusion

We conclude that the evidence for viral lateral transfer as the origin of microbial ORFans in general, is currently weak, and even negative. With better viral sampling the evidence will likely become stronger, but only for some of the clades, and only for a fraction of the ORFans. Even if lateral transfer from viruses turns out to be the main origin of microbial ORFans, one is still left with the need to explain the origin of the also abundant viral ORFans. In our viral genome database, 27% of the ORFs have no homologs (i.e. are viral ORFans) and only 20% of the ORFs have homologs in prokaryotes. Interestingly, by analyzing the functions of the viral homologs using COGs [29], we found that the percentage of ORFans' viral homologs that correspond to poorly characterized COGs (no COGs hits or hits to "Function unknown" or "General function prediction only") is much larger than that of the non-ORFans (87.2% vs. 49.5%). In any case, other alternative mechanisms may be required to explain the origin of ORFans, including among others, duplication followed by rapid divergence [12,30] or lateral transfer from non-viral organisms whose genomes have not been sequenced or which have since disappeared. Most likely, as is the case in so many cases in evolutionary biology, the origin of ORFans will turn out to be non-exclusive [31], and may include other yet-unknown mechanisms. Operating separately or in conjunction, these mechanisms may entail a more complete explanation of the puzzle of the origins of microbial and viral ORFans.

## Methods

### Building the ORFan dataset

We downloaded the 277 microbial genomes available at the NCBI [32] on Nov. 03, 2005. We carried out all vs. all (820,768 ORFs) BLASTP searches (masking the low complexity region in the query) to identify homologs for each ORF. 1,862 short ORFs composed mainly of low complexity regions were excluded from our database. Here we considered two proteins to be homologous if the BLASTP score was <1e-3 (for alignment lengths <80 we used 1e-5 instead) [2].

For each pair of genomes A and B, we calculated a similarity value of two genomes A and B as:

SIM(A,B)=NumberOfORFsInAHavingHomologsInBTotalORFsInA
 MathType@MTEF@5@5@+=feaafiart1ev1aaatCvAUfKttLearuWrP9MDH5MBPbIqV92AaeXatLxBI9gBaebbnrfifHhDYfgasaacH8akY=wiFfYdH8Gipec8Eeeu0xXdbba9frFj0=OqFfea0dXdd9vqai=hGuQ8kuc9pgc9s8qqaq=dirpe0xb9q8qiLsFr0=vr0=vr0dc8meaabaqaciaacaGaaeqabaqabeGadaaakeaacqqGtbWucqqGjbqscqqGnbqtcqGGOaakcqqGbbqqcqGGSaalcqqGcbGqcqGGPaqkcqGH9aqpdaWcaaqaaiabb6eaojabbwha1jabb2gaTjabbkgaIjabbwgaLjabbkhaYjabb+eapjabbAgaMjabb+eapjabbkfasjabbAeagjabbohaZjabbMeajjabb6gaUjabbgeabjabbIeaijabbggaHjabbAha2jabbMgaPjabb6gaUjabbEgaNjabbIeaijabb+gaVjabb2gaTjabb+gaVjabbYgaSjabb+gaVjabbEgaNjabbohaZjabbMeajjabb6gaUjabbkeacbqaaiabbsfaujabb+gaVjabbsha0jabbggaHjabbYgaSjabb+eapjabbkfasjabbAeagjabbohaZjabbMeajjabb6gaUjabbgeabbaaaaa@6DCB@

Notice that SIM(A, B) ≠ SIM(B, A). Notice also that similar measures have been used by others (e.g. [33,34]).

For each ORF, g, we calculated the value Hg as the number of genomes having at least one homolog of g, and Ug, a measure of the "uniqueness" of g as:

Ug=∑i=1Hg(1−SIM(A,Bi))Hg
 MathType@MTEF@5@5@+=feaafiart1ev1aaatCvAUfKttLearuWrP9MDH5MBPbIqV92AaeXatLxBI9gBaebbnrfifHhDYfgasaacH8akY=wiFfYdH8Gipec8Eeeu0xXdbba9frFj0=OqFfea0dXdd9vqai=hGuQ8kuc9pgc9s8qqaq=dirpe0xb9q8qiLsFr0=vr0=vr0dc8meaabaqaciaacaGaaeqabaqabeGadaaakeaacqqGvbqvcqqGNbWzcqGH9aqpdaWcaaqaamaaqahabaGaeiikaGIaeGymaeJaeyOeI0Iaem4uamLaemysaKKaemyta0KaeiikaGIaemyqaeKaeiilaWIaemOqaiKaemyAaKMaeiykaKIaeiykaKcaleaacqWGPbqAcqGH9aqpcqaIXaqmaeaacqWGibascqWGNbWza0GaeyyeIuoaaOqaaiabdIeaijabdEgaNbaaaaa@47C6@

where each B_i _corresponds to a genome having a homolog of g.

ORFs with H = 1 and one BLASTP hit were classified as singleton ORFans; ORFs with H = 1 and more than 1 BLASTP hits were classified as paralogous ORFans; ORFs with H > 1 and U <= 0.1 were classified as orthologous ORFans.

### Finding the virus homologs

We downloaded the Refseq release 13 (Sept. 2005) virus database from NCBI [35], containing 43,566 viral proteins cross referenced to 1456 NCBI taxonomical species. The 72 viral species in Refseq which do not encode proteins were not included in our analysis. We carried out BLASTP searches using each microbial protein as query against this dataset (masking with the low complexity filter). Homologs were defined as above.

### Statistical analysis

All two sample tests used are Wilcoxon nonparametric tests and are conducted by using R language [36].

## Authors' contributions

YY conducted the computation, analyzed data, and drafted the manuscript. DF designed the research, supervised this project and finalized the manuscript. All authors read and approved the final manuscript.

## Supplementary Material

Additional file 1 the numbers of ORFans and nonORFans, the numbers of ORFs having homologs in viruses in 277 prokaryotic genomes, using different BLASTP cutoff to define ORFans. Click here for file
